# Artificial cationic oligosaccharides for heteroduplex oligonucleotide-type drugs

**DOI:** 10.1038/s41598-018-22161-8

**Published:** 2018-03-12

**Authors:** Rintaro Iwata Hara, Yuki Hisada, Yusuke Maeda, Takanori Yokota, Takeshi Wada

**Affiliations:** 1 Faculty of Pharmaceutical Sciences, Tokyo University of Science, 2641 Yamazaki, Noda, Chiba 278-8510 Japan; 2 Graduate School of Frontier Sciences, The University of Tokyo, 5-1-5 Kashiwanoha, Kashiwa, Chiba 277-8562 Japan; 3 Course of Applied Life Science, Faculty of Applied Biological Sciences, Gifu University, 1-1 Yanagido, Gifu, 501-1193 Japan; 4 Department of Neurology and Neurological Science, Graduate School of Medical and Dental Sciences, Tokyo Medical and Dental University, 1-5-45, Yushima, Bunkyo-ku, Tokyo 113-8519 Japan

## Abstract

Heteroduplex oligonucleotides (HDOs), composed of a DNA/LNA gapmer and its complementary RNA, are a novel, promising candidates for antisense drugs. We previously reported oligodiaminogalactoses (ODAGals), designed to bind to A-type nucleic acid duplexes such as DNA/RNA and RNA/RNA duplexes. In this paper, we report oligodiguanidinogalactoses (ODGGals) as novel A-type duplex binding molecules. We aimed to study in detail applicability of ODAGals and ODGGals for additives to HDOs as an antisense drug. The effect of ODAGal4 (ODAGal 4mer) and ODGGal3 (ODGGal 3mer) on an HDO were evaluated by UV melting analyses, RNA degradation study by ribonuclease A (RNase A), and ribonuclease H (RNase H). Cleavage of a 13mer HDO by RNase A, which is considered to be the main cause of RNA degradation in serum, was effectively inhibited by the addition of only one equivalent of ODAGal4 and ODGGal3. In contrast, RNase H activity, which involves the cleavage of target RNAs by an antisense mechanism, was only slightly affected by the presence of the cationic oligosaccharides. These results suggest that ODAGal4 and ODGGal3 are useful because they could both stabilize the HDO and maintain RNase H activity of the gapmer.

## Introduction

Antisense oligonucleotides (ASOs) are among the most successful nucleic acid candidates. Four antisense drugs have been approved: fomivirsen (Vitravene^®^), mipomersen (Kynamro^®^), and eteplirsen (Exondys 51^TM^), nusinersen (Spinraza^®^)^1–4^. All these drugs are based on nucleic acids that have been chemically modified to be nuclease-resistant *in vivo* and have other favorable properties as drugs. The mechanisms of action of antisense drugs are mainly classified into two types^5^. In the first type, RNase H-dependent ASOs, which form a duplex structure with the complementary RNA and the RNA strand in the ASO/RNA duplex is cleaved by RNase H. The second type is steric blocker ASOs, which simply mask the function of the target RNA by duplex formation. In the former mechanism, the ASOs must contain consecutive DNA region for recognition by RNase H, as well as highly chemically modified nucleic acids to ensure the biological stability of the ASOs and the thermodynamic stability of the ASO/RNA duplex. One of the designs of effective RNase H-dependent ASOs is gapmer ASOs, which contain a central window of consecutive DNA and a wing region consisting of RNA-like nucleotides with high affinity for complementary RNA, such as 2′-OMe RNA and LNA^6^. Since gapmer type oligonucleotides was reported to show RNase H activity in 1980s^7,8^, they have been eagerly studied as nucleic acid drug candidates^2,6,9^ and the mipomersen is a successful one. Recently, novel gapmer-based nucleic acid drug candidates called heteroduplex oligonucleotides (HDOs) have been reported^10^. An HDO is composed of an LNA/DNA/LNA gapmer and its complementary RNA. The gapmer is also fully modified with phosphorothioate (PS); the complementary RNA contains partial PS and 2′-OMe modifications on a few nucleotides at each terminus, and the α-tocopherol moiety is anchored to the 5′-end for effective liver delivery.

**Figure 1:**
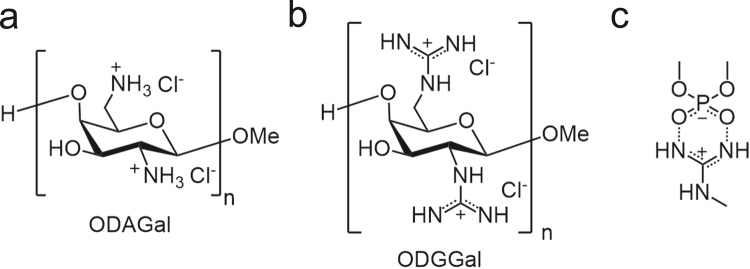
Structure of ODAGals (**a**) and ODGGals (**b**), and the interaction of guanidino groups with phosphate groups by two hydrogen bonds (**c**).

We have reported cationic oligosaccharides that bind to A-type nucleic acid duplexes such as RNA/RNA duplexes and DNA/RNA hybrids. In a recent study, we reported the synthesis of β-(1 → 4)-linked 2,6-diamino-2,6-dideoxy D-galactopyranose oligomers (ODAGals, oligodiaminogalactoses), and showed that the ODAGal 4mer (ODAGal4) could effectively bind to an siRNA and protect it almost completely against cleavage by RNase A^11^. In addition, ODAGals can be conjugated with ligand molecules for effective drug delivery systems of nucleic acid drugs, and synthesis of ODAGal-vitamin E conjugates was previously reported for liver delivery of RNAi drugs^12^. One of the most distinctive feature of ODAGal derivatives is that they can bind to major groove of A-type nucleic acid duplexes with high affinity and selectivity. From these properties, ODAGal4 could protect an siRNA with low N/P ratio even less than 1 whereas an excess amount of conventional cationic molecules was required to show same protection ability (N indicates cations and P does phosphate and phosphorothioate anions). In this study, we report the synthesis of β-(1 → 4)-linked 2,6-dideoxy-2,6-diguanidino-D-galactopyranose oligomers (ODGGals, oligodiguanidinogalactoses, Fig. 1b). Guanidino groups are known to effectively interact with phosphate groups by two hydrogen bonds (Fig. 1c)^13^. We evaluated the binding properties of ODGGals to nucleic acid duplexes, and the effect of ODGGals and ODAGals on the RNase A resistance of an HDO and the RNase H activity of the gapmer.

## Results

### Synthesis of ODGGals

**Figure 2:**
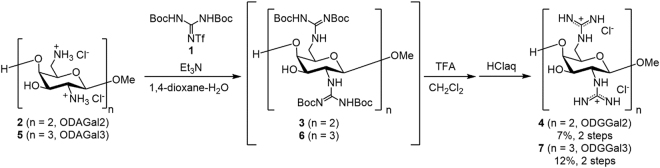
Synthesis of ODGGals.

Synthesis of the ODGGals was conducted according to Fig. 2 by transforming the amino groups in the ODAGals^11^. First, ODAGal2 **2** and ODAGal3 **5** were guanidinylated using Goodman’s reagent^14^. In both cases, because ODAGals are hydrophilic molecules and compounds **3** and **6** are hydrophobic molecules, it was necessary to add 1,4-dioxane portionwise in parallel with the reaction progress. After guanidinylation, the Boc groups were removed under acidic conditions^15^ to afford ODGGal2 **4** and ODGGal3 **7** in yields of 7% and 12% yields, respectively. The low yields were mainly attributed to incomplete guanidinylation even under optimized conditions.

### UV melting analyses

To evaluate the effects of ODGGal3 on the thermodynamic stability of nucleic acid duplexes, UV melting analyses were performed using RNA/RNA duplexes, a DNA/RNA hybrid, and DNA/DNA duplexes (Table 1). First, the melting temperature of a self-complementary RNA/RNA duplex, r(CGCGAAUUCGCG)_2_, and the ∆*T*_m_ values in the presence of the ODGGal 2mer or ODGGal3 were determined, as shown in Entry 1. The ODGGal 2mer slightly increased the *T*_m_ value (2.3 °C), and the ∆*T*_m_ value in the presence of ODGGal3 was significantly high (13.2 °C). Next, Entries 2 to 5 show the *T*_m_ and ∆*T*_m_ values of other RNA/RNA duplexes, a DNA/RNA hybrid, and a DNA/DNA duplex in the absence and presence of ODGGal3. For the RNA/RNA duplexes, the ∆*T*_m_ values were greater than 10 °C in all cases although the ∆*T*_m_ value in Entry 3 could not be precisely calculated because the *T*_m_ value was too high. The base sequence of DNA/RNA in Entry 4 corresponds to the RNA/RNA duplex in Entry 2, and the ∆*T*_m_ value in Entry 4 (7.9 °C) was lower than that of the RNA/RNA duplex (11.9 °C). As shown in Entry 5, the ∆*T*_m_ value of the DNA/DNA duplex in the presence of ODGGal3 was relatively low (3.8 °C) compared to those of the RNA/RNA and DNA/RNA duplexes. While these tendencies the ODGGal3 were quite similar to those of the ODAGals in our previous report, all the ∆*T*_m_ values were significantly higher than not only those of the ODAGal3, but also those of the ODAGal4^11^. These results suggest that the replacement of the amino groups by guanidino groups was effective to strengthen the thermostabilizing abilities of the ODAGals for A-type nucleic acid duplexes. We also conducted fluorescence anisotropy measurements to evaluate the binding affinity of ODGGal3 to these nucleic acid duplexes and the *K*_d_ values are shown in the Supplementary Information (Table S1). ODGGal3 were found to bind to the A-type nucleic acid duplexes in the *K*_d_ values of 4.9 × 10^−8^-1.6 × 10^−7^ M and not bind so strongly to the DNA/DNA duplex.

**Table 1:** *T*_m_ and ∆*T*_m_ values of nucleic acid duplexes in the absence and presence of ODGGals.

Entry	duplex	*T*_m_/°C	*∆T*_m_ (+1 equiv)/°C	
			ODGGal2	ODGGal3
1	RNA/RNA	64.2	2.3	13.2
2	RNA/RNA	60.6		11.9
3	RNA/RNA	77.6		>10
4	DNA/RNA	48.0		7.9
5	DNA/DNA	53.2		3.8

Base sequences are as follows: Entry 1: CGCGAAUUCGCG/CGCGAAUUCGCG, Entry 2: (CAGU)_3_/(ACUG)_3_, Entry 3: AACCCGCGGGUU/AACCCGCGGGUU, Entry 4: (cagt)_3_/(ACUG)_3_, Entry 5: cgcgaattcgcg/cgcgaattcgcg. B (capital) = RNA; b (lower case) = DNA.

Next, the *T*_m_ values of the HDO and the gapmer/RNA duplex (“RNA” indicates chemically unmodified RNA) in the absence and presence of ODGGal3 or ODAGal4, were measured. As shown in Table 2, the ∆*T*_m_ values (13.3 °C for ODGGal3 and 8.3 °C for ODAGal4) were more similar to those of the RNA/RNA duplexes than those of the DNA/RNA duplexes in Table 1; the HDO is expected to have the structural features of both DNA/RNA hybrids and RNA/RNA duplexes. As shown in Entry 2, the ∆*T*_m_ values of the gapmer/RNA duplex (13.9 °C for ODGGal3 and 7.8 °C for ODAGal4) were almost the same as those of the HDO.

**Table 2:** *T*_m_ and ∆*T*_m_ values of the HDO and the gapmer/RNA hybrid in the absence and presence of ODGGals.

Entry	duplex	*T*_m_/°C	*∆T*_m_ (+1 equiv)/°C	
			ODAGal4	ODGGal3
1	HDO	56.1	8.3	13.3
2	gapmer/RNA	58.7	7.8	13.9

Base sequences and chemical modifications are as follows; HDO: G^L^_s_mC^L^sa_s_t_s_t_s_g_s_g_s_t_s_a_s_t_s_T^L^_s_mC^L^_s_A^L^/Toc-U^M^_s_G^M^_s_A^M^_s_AUACCAAU_s_G^M^_s_C^M^, gapmer/RNA: G^L^_s_mC^L^sa_s_t_s_t_s_g_s_g_s_t_s_a_s_t_s_T^L^_s_mC^L^_s_A^L^/UGAAUACCAAUGC. B^L^ = LNA; B^M^ = 2′-OMe RNA; B_s_ or b_s_ indicates a PS modified nucleotide; mC indicates 5-methyl-C; Toc indicates α-tocopherol.

### Effects on RNase A resistance of a DNA/RNA hybrid and HDO

**Figure 3:**
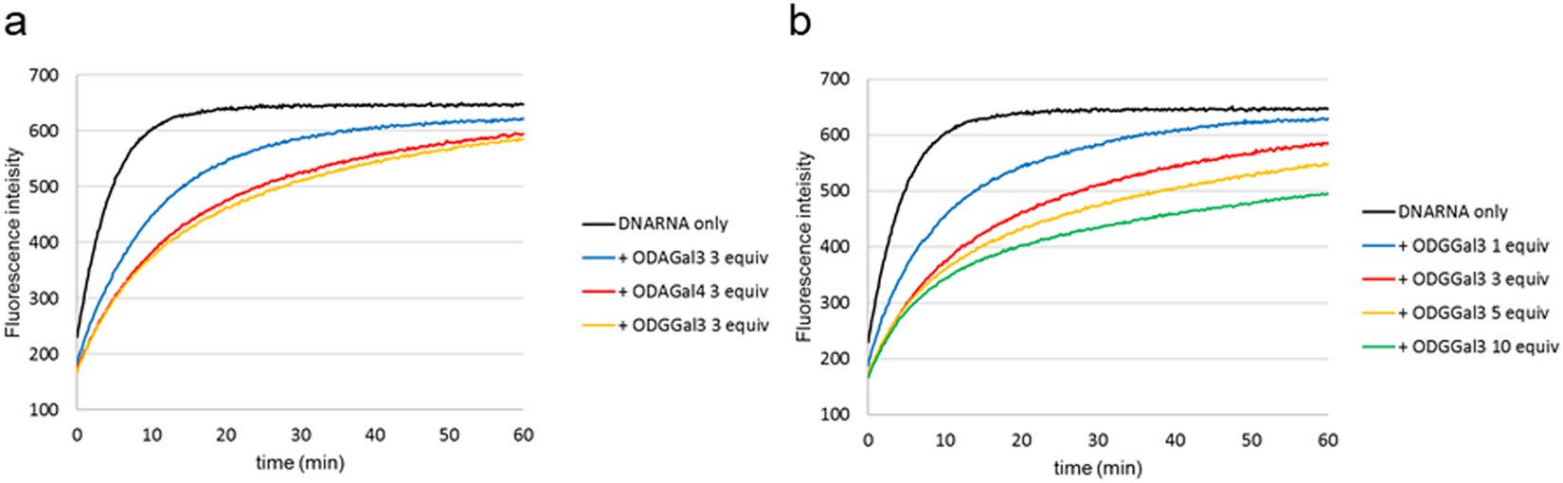
Fluorescence signals as a function of time to study the RNase A resistance of a DNA/RNA hybrid: (**a**) in the presence of three equivalents of cationic oligosaccharides. (**b**) In the presence of various equivalent of ODGGal3. The base sequence and modifications are as follows; (cagt)_3_/FAM-(ACUG)_3_-Dabcyl. The hybrid was treated with 0.5 μg/mL of RNase A at 30 °C, pH = 7.3. The initial concentration of the hybrid was 100 nM in a Tris-HCl buffer containing 100 mM NaCl.

To evaluate RNase A resistances, a fluorescence resonance energy transfer (FRET) system was applied with a FAM group and a Dabcyl group^16^. First, the RNase A resistance of the DNA/RNA hybrid was evaluated with DNA and FAM- and Dabcyl-modified RNA in the absence and presence of ODAGal3, ODAGal4, or ODGGal3. In these FRET systems, an increase in the fluorescence intensity reflects cleavage of the RNA strand; as shown in Fig. 3(a), the fluorescence intensity increased upon addition of RNase A and became almost saturated after approximately 20 min in the absence of cationic saccharides. In contrast, three equivalents of ODAGal3, ODAGal4, and ODGGal3 all showed inhibition abilities against RNase A degradation of the RNA strand in the DNA/RNA hybrid. The inhibition abilities of ODAGal4 and ODGGal3 were similar and both cationic molecules were more effective than ODAGal3. These results showed that both an increase in the number of cationic molecules and the replacement of amino groups with guanidino groups improved the protection of the RNA strand in a DNA/RNA hybrid against RNase A degradation.

Figure 3(b) shows the dose dependency of ODGGal3. As the amount of ODGGal3 increased, the rate of increase of the fluorescence intensity apparently slowed. However, even in the presence of ODGGal3 with a concentration 10 times higher than that of the DNA/RNA hybrid, inhibition of the fluorescence increase was moderate. These results suggest that it is difficult to protect the hybrid completely against RNase A degradation in contrast to the siRNA results in our previous study^11^, in which an siRNA was almost completely protected against RNase A by the addition of only three equivalents of ODAGal4.

**Figure 4:**
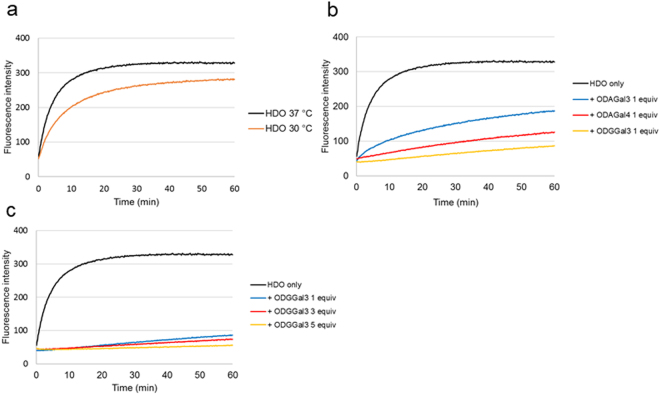
Fluorescence signals as a function of time to study the RNase A resistance of the HDO: (**a**) in the absence of cationic saccharides at 30 °C and 37 °C. (**b**) In the presence of one equivalent of cationic oligosaccharides at 37 °C. (**c**) In the presence of various equivalents of ODGGal3 at 37 °C. The base sequence and modifications are as follows; Dabcyl-G^L^_s_mC^L^_s_a_s_t_s_t_s_g_s_g_s_t_s_a_s_t_s_T^L^_s_mC^L^_s_A^L^/Toc-U^M^_s_G^M^_s_A^M^_s_AUACCAAU_s_G^M^_s_C^M^-FAM. The HDO was treated with 0.5 μg/mL RNase A at 30 °C, pH 7.3 or 37 °C, pH 7.2. The initial concentration of the hybrid was 100 nM in a Tris-HCl buffer containing 100 mM NaCl.

Next, we applied these systems to the HDO. 5′-Dabcyl-modified-gapmer and 3′-FAM-modified-RNA were used in these experiments. As shown in Fig. 4(a), the RNA strand in the HDO was found to be less susceptible to RNase A; the fluorescence intensity did not become saturated in 60 min at 30 °C, the same conditions used for the DNA/RNA duplex. Therefore, the susceptibility of the HDO to RNase A was evaluated at 37 °C; at this temperature, the fluorescence intensity became saturated in 30 min in the absence of cationic oligosaccharides. In contrast, the cationic oligosaccharides significantly inhibited this increase (Fig. 4(b)). In particular, ODGGal3 was demonstrated to be significantly effective in protecting the HDO from cleavage by RNase A; the change in fluorescence intensity was very slow in the presence of even one equivalent of ODGGal3.

Furthermore, fluorescence-intensity increase rate became increasingly slow as the amount of ODGGal3 was increased, as shown in Fig. 4(c). In the presence of five equivalents of ODGGal3, almost no increase in fluorescence-intensity was observed in 60 min. These results strongly suggested that the cleavage of RNA strand in the HDO could be significantly suppressed by addition of ODGGal3. Furthermore, it was also suggested by comparing Fig. 4 with Fig. 3 that the combination of chemical modifications in the HDO and ODGGal3 could lead high RNase A resistance, which neither only chemical modifications nor only cationic oligosaccharides could accomplish.

### RNase H activity of the gapmer

**Figure 5:**
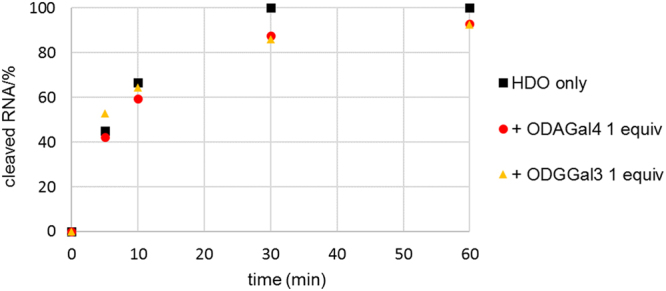
Amounts of cleaved RNA at various reaction times after treatment with RNase H. 4 μM of the gapmer/RNA duplex (i.e., G^L^_s_mC^L^_s_a_s_t_s_t_s_g_s_g_s_t_s_a_s_t_s_T^L^_s_mC^L^_s_A^L^/UGAAUACCAAUGC) was treated with 40 U/mL of RNase H in 10 mM Tris buffer containing 100 mM NaCl and 0.5 mM of MgCl_2_ at pH 7.2 and 37 °C.

**Figure 6:**
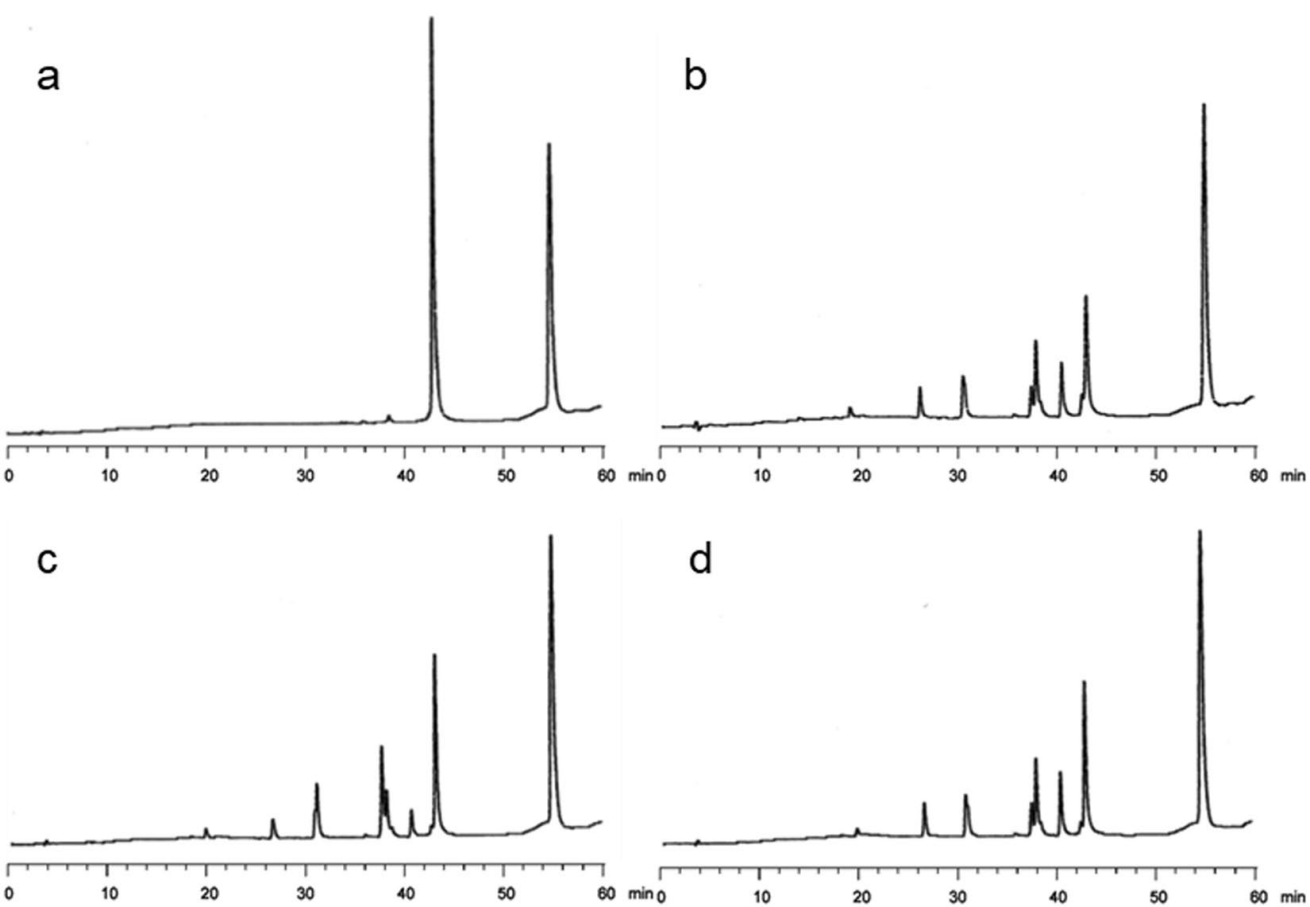
(**a**) HPLC profiles of the gapmer/RNA duplex. (**b**) to d) HPLC profiles of the gapmer/RNA duplex after treatment with 40 U/mL of RNase H over 10 min at 37 °C: (**b**) in the absence of cationic molecules: (**c**) in the presence of ODAGal4 (1 equiv): (**d**) in the presence of ODGGal3 (1 equiv).

Finally, we evaluated the effect of ODAGal4 and ODGGal3 on the RNase H activity of the LNA-DNA-LNA gapmer in the HDO. In these experiments, after treatment of a duplex of the gapmer and the complementary RNA 13mer with RNase H for 5 to 60 min in the absence and presence of cationic oligosaccharides, the amount of intact RNA was evaluated by RP-HPLC analysis. The amount of intact RNA 13mer lost under each condition are plotted in Fig. 5. The cleavage of the RNA by RNase H was demonstrated to proceed at a similar rate regardless of the absence or presence of ODAGal4 or ODGGal3. The typical RNA degradation profiles are shown in Fig. 6. The same degradation products were generated by cleavage in both the absence and presence of the cationic saccharides although the respective amounts of the fragments were different.

**Figure 7:**
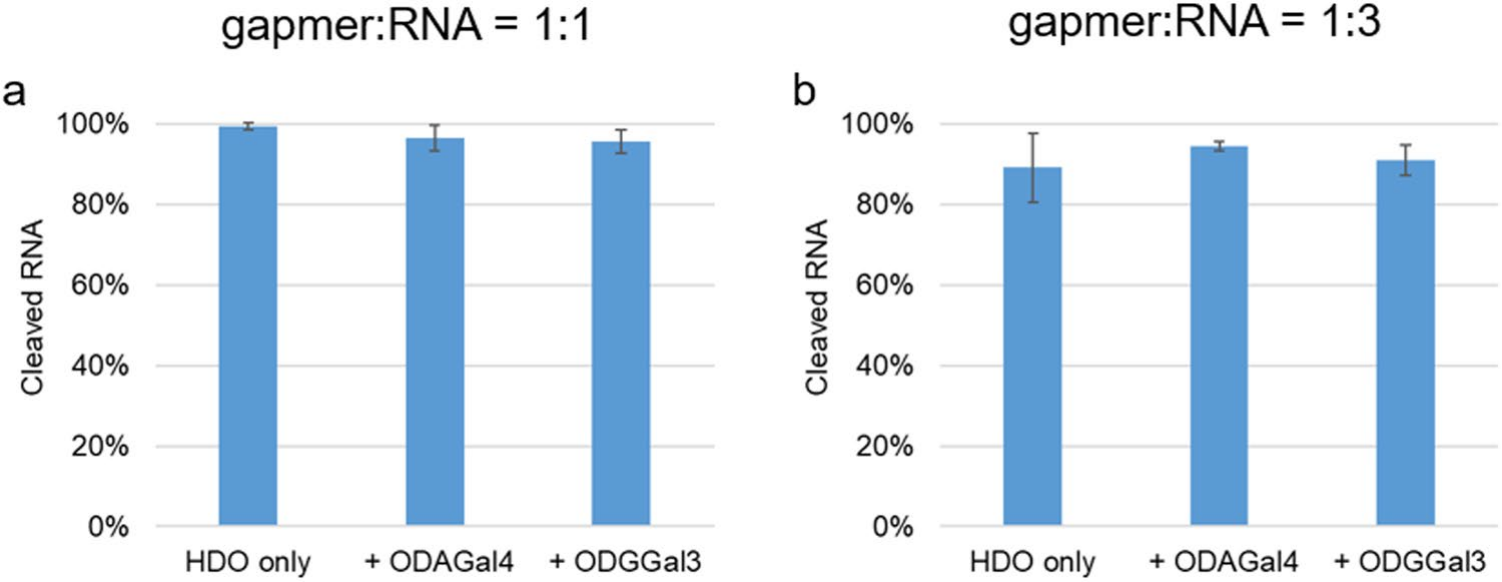
Amounts of cleaved RNA after treatment with RNase H for 1 h. The mixture of 4 μM of the gapmer and 4 or 12 μM of the complementary RNA was treated with 40 U/mL of RNase H in 10 mM Tris buffer containing 100 mM NaCl and 0.5 mM of MgCl_2_ at pH 7.2 and 37 °C. Student t test was used to statistical significance of the results. n = 3. (**a**) 4 μM of the gapmer and 4 μM of the complementary RNA.; SD = 0.8% (HDO only), 3.2% (+ODAGal4), 1.7% (+ODGGal3), p = 0.293 (HDO only/+ODAGal4), 0.204 (HDO only/+ODGGal3). (**b**) 4 μM of the gapmer and 12 μM of the complementary RNA.; SD = 8.7% (HDO only), 1.2% (+ODAGal4), 3.8% (+ODGGal3), p = 0.343 (HDO only/+ODAGal4), 0.597 (HDO only/+ODGGal3).

We further conducted the RNA degradation experiments using the RNA in three times amount of the gapmer. Under the reaction conditions, the catalytic reaction efficiency could be evaluated because two-thirds of the RNA strand initially do not form a duplex. The reaction time was 1 h, which is enough to cleave the RNA strand which initially formed duplex to the gapmer (also see Fig. 5). As shown in Fig. 7b, more than 90% of the intact RNA was cleaved even in the presence of ODAGal4 or ODGGal3. It was clearly demonstrated from these results that these cationic oligosaccharides did not affect the rate of the catalytic reaction of RNase H including the release and re-cleavage of RNA strands.

## Discussion

RNA/RNA duplexes are known to form A-type duplex structures^17,18^, and DNA/RNA hybrids form duplexes between A-type and B-type^19,20^. ODAGal4 and other cationic oligosaccharides have been reported to favor RNA/RNA duplexes over DNA/RNA hybrids and far over DNA/DNA duplexes^11^. We have explained this binding selectivity on the basis of the major groove width of the duplexes. The major groove of A-type RNA/RNA duplexes is known to be narrower than that of B-type DNA/DNA duplexes^18,20^. The distance between the amino groups in the 2- and 6-positions of ODAGal4 is appropriate to bind to A-type duplexes and too short to bind to B-type duplexes. Although the structure of DNA/RNA hybrids are closer to A-type than B-type, major groove widths of the hybrids tend to be wider than RNA/RNA duplexes in the NMR structures^19^. In addition, there might be differences in the flexibility of a DNA strand and an RNA strand. The DNA strand in the hybrids is more flexible than the RNA strand. Binding of the cationic oligosaccharide probably decrease the flexibility, and these differences might also explain the reason why the cationic oligosaccharides favor RNA/RNA duplexes over DNA/RNA hybrids. From these viewpoints, it is important whether the effects of the ODAGal4 addition to the HDO is similar to those to RNA/RNA (LNA/RNA) or DNA/RNA duplexes. From the UV melting analyses, the increases in the *T*_m_ values of the HDO upon addition of ODAGal4 or ODGGal3 were close to those of the RNA/RNA duplexes. We measured CD spectra of the HDO in the absence and presence of the cationic oligosaccharides and it was found that they were typical ones of A-type duplexes in all cases (Supplementary Information, Figure S18)^21,22^. These results strongly suggested that the structure of the HDO is closer to an A-type structure than an A-B-type structure due to by LNA residues in the terminuses whereas the HDO must have enough flexibility in the DNA region to form an A-B type structure because the gapmer shows RNase H activity;^6,23^ In addition, when discussing this topic, it should be noted that only four or five phosphate groups at the 5′ terminus in each strand exist in the major groove in 13mer/13mer duplexes. The tertiary structure in the wing region and its neighborhood in the HDO is expected to be a rigid A-type structure due to LNA residues in the termini; *i.e*., the major groove, the binding site of ODAGal4 and ODGGal3 in the HDO, can form an A-type duplex structure, and these cationic oligosaccharides effectively stabilize the duplex. Another consideration is PS modifications. The PS linkages in the HDO are also expected to affect its interactions with ODAGal4 and ODGGal3 because the sulfur atoms in place of the oxygen atoms can interact with amino or ganidino groups of these cationic oligosaccharides. However, the difference between the ∆*T*_m_ value of the HDO in which the RNA strand was partially modified with PS linkages and that of the gapmer/all-PO-RNA is quite small in the presence of cationic oligosaccharides. On the basis of this comparison, we suggest that the ∆*T*_m_ values are mainly derived from structural features rather than PS modifications. We previously reported a cationic oligosaccharide more thermodynamically stabilized an all-(*R*p)-PS-DNA/RNA duplex than a PO-DNA/RNA duplex. In the report, however, we used stereoregulated PS-DNA (*i.e*. an all-(*S*p)-PS DNA and an all-(*R*p)-PS DNA)^24^. On the other hand, Arya *et al*. reported PS-linkage not much affected the binding ability of aminoglycosides and their stabilizing effect to DNA/RNA duplexes using stereorandom PS-DNA a year after our report^25^. In this study, we used the gapmer and Toc-RNA having stereorandom PS linkages and therefore the effect of the PS-modifications might be small in the UV melting analyses.

RNase A is an endonuclease that cleaves single-stranded RNA. In addition, RNase A is known to cleave RNA strands in duplex structures; in previous studies, the cleavage of siRNAs in serum was reported to be mainly due to endogenous RNase A-like nucleases^26,27^. In the case of siRNAs, it was reported that a less thermodynamically stable duplex was more susceptible to RNase A cleavage; this may be because a partially dissociated region in the duplex can be recognized by RNase A^26^. Our previous report regarding siRNA-ODAGal4 complex supported this cleavage mechanism; the siRNAs were almost completely protected against RNase A even in anion-excess systems^11^. In DNA/RNA and HDO, cleavage of the RNA strand should occur by the same mechanism as that of siRNAs. In contrast, in the current study, the cleavage of the RNA strand in the DNA/RNA hybrid was not effectively inhibited even in the presence of large amounts of cationic oligosaccharides. Although the oligomer length and original *T*_m_ value were different in both cases, the difference in the degree of increase in RNase A resistance in the presence and absence of cationic oligosaccharides may arise from the thermodynamic stabilization effect of the duplex structure in the unstable terminus by the cationic saccharides in both cases. Therefore, discussion of RNase A resistance based on *T*_m_ values is important although the ∆*T*_m_ values may not completely correspond with RNase A resistance because the *T*_m_ value provides an index of the thermodynamic stability of the entire structure of a duplex. In the case of the HDO, effective inhibition of RNase A cleavage was observed even upon addition of one equivalent of ODAGal4 or ODGGal3 (i.e. the N/P ratios are under 0.4 in both cases). This effective protection may be at least partly due to significant thermodynamic stabilization of HDO by the cationic oligosaccharides. Furthermore, these results also suggest the formation of an HDO-ODAGal4 or HDO-ODGGal3 1-to-1 complex at a low concentration of 100 nM at 37 °C; this is promising for *in vivo* applications in the foreseeable future.

RNase H is an enzyme that specifically recognizes DNA/RNA hybrids and cleaves the RNA strand. In this study, the RNase H activity of the LNA-DNA-LNA gapmer was almost unchanged upon addition of ODAGal4 or ODGGal3. This is clearly a contrasting result, considering that these cationic oligosaccharides effectively inhibit RNase A cleavage. These results are promising for antisense drug application because it was demonstrated that ODAGal4 and ODGGal3 could increase the thermodynamic stability and RNase A resistance without the inhibition of catalytic cleavage of RNA strands by RNase H. In addition, it is interesting to compare these results with a previous report in which paromomycin, a naturally occulting amino sugar, was reported to inhibit RNase H rather than RNase A cleavage of DNA/RNA hybrids^28^. To explain our desirable results for nucleic drug applications, we should consider the binding mode of RNase H and cationic oligosaccharides to the gapmer/RNA hybrid. ODAGal4 and ODGGal3 are expected to bind to some phosphodiester or thiophosphodiester groups at the 5′-teminus that form the major groove as previously stated. In contrast, on the basis of crystal structures of RNase H-DNA/RNA complexes^29,30^ and studies of the RNase H cleavage sites in DNA/RNA hybrids^7,31^, phosphate groups closer to the 3′ terminus in both the DNA and RNA strands of DNA/RNA complexes have been suggested to be important in RNase H recognition. For this reason, binding of the cationic oligosaccharides and RNase H may be not competitive in the 13mer HDO. This hypothesis is also supported by finding that the kind of RNA fragments did not vary upon addition of cationic oligosaccharides.

## Conclusion

In this study, we evaluated the effects of artificial cationic oligosaccharides, ODAGal4 and ODGGal3. These oligosaccharides were demonstrated to effectively protect an HDO against RNase A cleavage without decreasing RNase H activity of the gapmer. These results suggest that ODAGal4 and ODGGal3 are promising additives for internal administration of HDOs. *In vivo* assays and further experiments regarding nucleic drug applications are currently in progress.

## Methods

### General information

^1^H NMR spectra were obtained on a 300 MHz Varian MERCURY 300 spectrometer with acetonitrile as an internal or external standard (δ 2.06) in D_2_O. Mass spectra were recorded on a Voyager 4327 system (Applied Biosystems) or a 910-MS FTMS system (Agilent Technologies). Purification by prep. TLC was conducted using Merck Kieselgel 60 F_254_ silica gel. The organic solvents were purified and dried using appropriate procedures. The yields of the compounds were estimated from the integral values in the NMR spectra compared with acetonitrile (internal standard). DNA, RNA, and other nucleic acid oligomers were purchased from Japan Bio Services Co., Ltd., Hokkaido System Science Co., Ltd., or GeneDesign, Inc.

### Synthesis of compounds

#### Compound **4** (ODGGal2)

Goodman’s reagent **1** (150 mg, 383 μmol) and compound **2** (140 μmol) were dissolved in a solvent mixture of H_2_O and 1,4-dioxane (1:1, v/v, 1.0 mL). To this solution, triethylamine (150 μL) was added. After 16 h 1,4-dioxane (0.25 mL) was added; after an additional 73 h, more 1,4-dioxane (0.5 mL) was added. After 71 h, the solution was diluted with chloroform and washed with 10% aqueous citric acid solution. The organic layer was dried over Na_2_SO_4_, filtered, and concentrated. The crude product was purified by prep. TLC (dichloromethane-methanol (9:1, v/v)) to afford compound **3** containing some impurities not derived from sugar compounds (26.1 mg). 10 mg of the crude product was dissolved in a solvent mixture of TFA-dichloromethane (1:1, v/v, 4 mL). After 70 min, toluene (2 mL) was added and the solution was concentrated. The mixture was dissolved in 0.1 M hydrochloric acid (1.5 mL) and concentrated; this procedure was repeated four times. Then, the mixture was dissolved in water (1.5 mL) and concentrated; this procedure was repeated three times. The crude product was purified by precipitation (methanol-diethyl ether) to afford pure **4** (3.7 μmol, 7%, 2 steps).

^1^H NMR (300 MHz, D_2_O) δ 4.80–4.74 (m, 1 H), 4.48 (d, *J* = 8.4 Hz, 1 H), 4.17 (s, 1 H), 4.01–3.95 (m, 2 H), 3.90–3.79 (m, 3 H), 3.72–3.40 (m, 8 H), 3.31 (t, *J* = 9.0 Hz, 1 H).

ESI-MS: calcd for C_17_H_37_N_12_O_7_ m/z [M+H]^+^: 521.29 Found: 521.29.

#### Compound **7** (ODGGal3)

Goodman’s reagent **1** (550 mg, 1.44 mmol) and compound **5** (80 μmol) were dissolved in a solvent mixture of H_2_O and 1,4-dioxane (1:1, v/v, 1.0 mL). To this solution, triethylamine (200 μL) was added. After 17 h, 1,4-dioxane (0.25 mL) was added; after an additional 8 h, more 1,4-dioxane (0.25 mL) was added. After 22 h 1,4-dioxane (0.5 mL) was added; after an additional 74 h, more 1,4-dioxane (0.5 mL). After 7 d, the solution was diluted with dichloromethane and washed two times with 10% aqueous citric acid solution. The aqueous layer was back-extracted with dichloromethane two times; then, the combined organic layers were dried over Na_2_SO_4_, filtered, and concentrated. The crude product was purified by prep. TLC (dichloromethane-methanol (9:1, v/v)) to afford compound **6** containing some impurities not derived from sugar compounds (33 mg). 17 mg of the crude product was dissolved in a solvent mixture of TFA-dichloromethane (1:1, v/v, 6 mL). After 2 h, toluene was added and the solution was concentrated. The mixture was dissolved in 0.1 M hydrochloric acid (1 mL) and concentrated; this procedure was repeated four times. Then the mixture was dissolved in water (1 mL) and concentrated; this procedure was repeated four times. The crude product was purified by precipitation (methanol-diethyl ether) to afford pure **7** (4.7 μmol, 12%, 2 steps).

^1^H NMR (D_2_O, 300 MHz) δ 4.80–4.74 (m, 2 H), 4.49 (d, *J* = 8.4 Hz, 1 H), 4.16–4.11 (m, 2 H), 4.01–3.94 (m, 3 H), 3.88–3.78 (m, 4 H), 3.71–3.53 (m, 7 H), 3.50–3.26 (m, 5 H).

MALDI-TOF MS: calcd for C_25_H_53_N_18_O_10_ m/z [M + H]^+^: 765.42 Found: 765.50.

### UV melting analyses

Absorbance versus temperature profile measurements were conducted with an eight-sample cell changer in quartz cells with a path length of 1 cm. All the experiments were conducted in 10 mM phosphate buffer containing 100 mM NaCl at pH 7.0. The concentration of the duplex was 5 μM. The UV absorbance at both 260 and 320 nm was monitored with the temperature. The samples containing oligonucleotides were first rapidly heated to 95 °C, maintained at this temperature for 20 min, and finally allowed to cool to room temperature at a rate of 0.5 °C/min. ODAGal or ODGGal was added to the solution; these samples were additionally cooled to 0 °C or 20 °C at a rate of 0.5 °C/min, left at this temperature for 10 min. Finally, the dissociation was recorded by heating the samples to 95 °C at a rate of 0.5 °C/min.

### Evaluation of RNase A resistance

All experiments were conducted using 100 nM DNA/RNA or HDO and 0 to 1000 nM of ODGGal or ODAGal in 10 mM Tris buffer containing 100 mM NaCl, pH 7.2 and 37 °C or pH 7.3 and 30 °C. To a solution of duplex with or without cationic oligosaccharides, 100 μg/mL of RNase A from bovine pancreas in Tris buffer was added to afford a final concentration of 0.50 μg/mL. 10 s after addition of RNase A, the fluorescence intensity was recorded (10 s intervals) for 3600 s. The following instrument settings were used: Ex/Em, 490 nm/520 nm; response, 1 sec; band width (Ex), 1 nm; band width (Em), 3 nm.

### Evaluation of RNase H activity

All experiments were conducted using 4 μM gapmer, 4 or 12 μM RNA and 4 μM ODAGal4 or ODGGal3 in 10 mM Tris buffer containing 100 mM NaCl and 0.5 mM of MgCl_2_ at pH 7.2 and 37 °C. To the solution of the gapmer/RNA hybrid with or without cationic oligosaccharides, 500 U/mL of RNase H from *E. coli* in Tris buffer was added to afford a final concentration of 40 U/mL. After 5, 10, 30, and 60 min, respectively, 30 μL of the solution was divided from the reaction mixture and heated at 95 °C for 3 min. Then the solution was cooled and analyzed by reversed-phase HPLC (RP-HPLC). RP-HPLC was conducted using a μ Bondasphere 5 μm C18 column, 100 Å, 3.9 mm × 150 mm with a linear gradient of 0% to 10% acetonitrile over 45 min followed by 10% to 40% acetonitrile over 15 min in 0.1 M TEAA buffer at 50 °C at a rate of 0.5 mL/min or a linear gradient of 0% to 10% acetonitrile over 30 min followed by 10% to 50% acetonitrile over 10 min in 0.1 M TEAA buffer at 50 °C at a rate of 0.5 mL/min.

## Electronic supplementary material

Supplementary Information
