# Supplementary material for: Artificial cationic oligosaccharides for heteroduplex oligonucleotide-type drugs
Source: Sci Rep. 2018 Mar 12;8:4323. doi: 10.1038/s41598-018-22161-8 (PMC5847598; doi:10.1038/s41598-018-22161-8)
Supplement: Supplementary file 1 — Supplementary Information [file 41598_2018_22161_MOESM1_ESM.pdf]

Supplementary Information for

# Artificial cationic oligosaccharides for heteroduplex oligonucleotide type drugs

Rintaro Iwata Hara[1], Yuki Hisada[2], Yusuke Maeda[3], Takanori Yokota[4] and Takeshi Wada[1,*]

*[1] Faculty of Pharmaceutical Sciences, Tokyo University of Science, 2641 Yamazaki, Noda, Chiba 278-8510, Japan.*

*[2] Graduate School of Frontier Sciences, The University of Tokyo, 5-1-5 Kashiwanoha, Kashiwa, Chiba 277-8562, Japan.*

*[3] Course of Applied Life Science, Faculty of Applied Biological Sciences, Gifu University, 1-1 Yanagido, Gifu, 501-1193, Japan.*

*[4] Department of Neurology and Neurological Science, Graduate School of Medical and Dental Sciences, Tokyo Medical and Dental University, 1-5-45, Yushima, Bunkyo-ku, Tokyo, 113-8519, Japan*

*E-mail:* twada@rs.tus.ac.jp

Table of contents

NMR spectra 2

UV melting curves 3

Fluorescence anisotropy measurements 7

HPLC profiles after treatment with RNase H 10

CD spectra 13

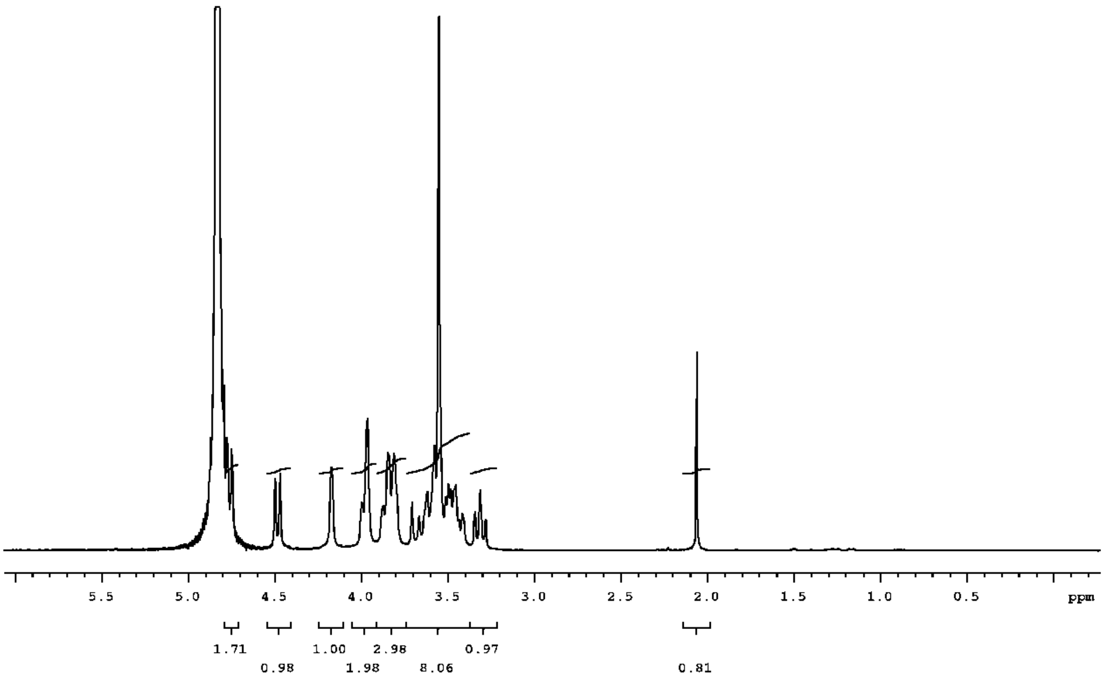


Figure S1. $^{1}$H NMR spectrum of compound ODGGal2 **4** in $D_2O$

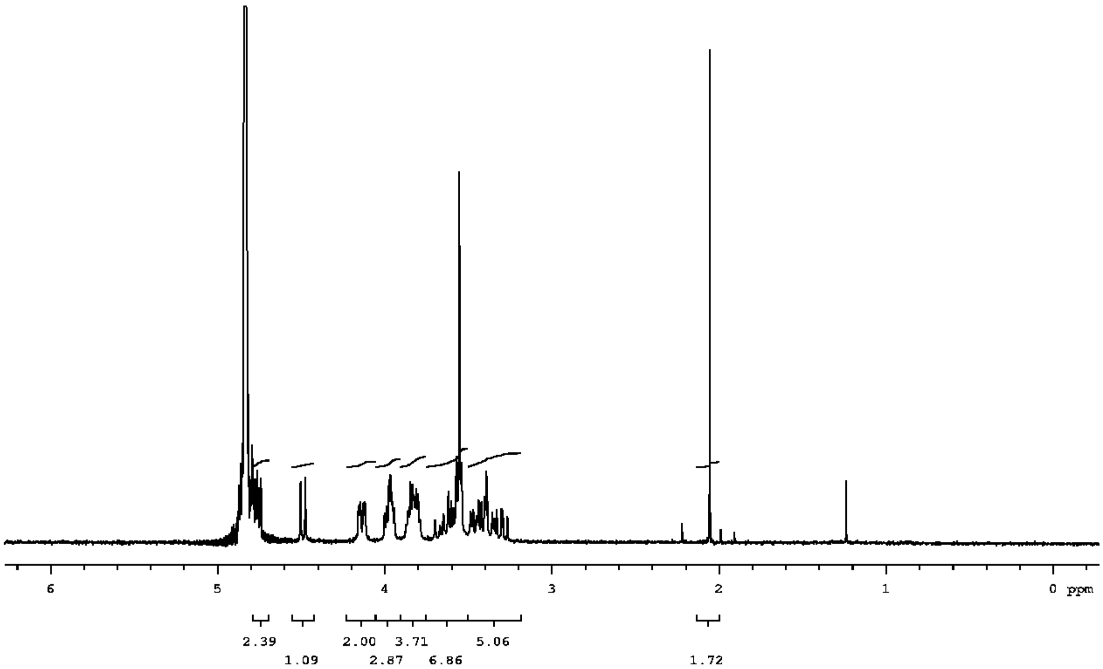


Figure S2. $^{1}$H NMR spectrum of compound ODGGal3 **7** in $D_2O$

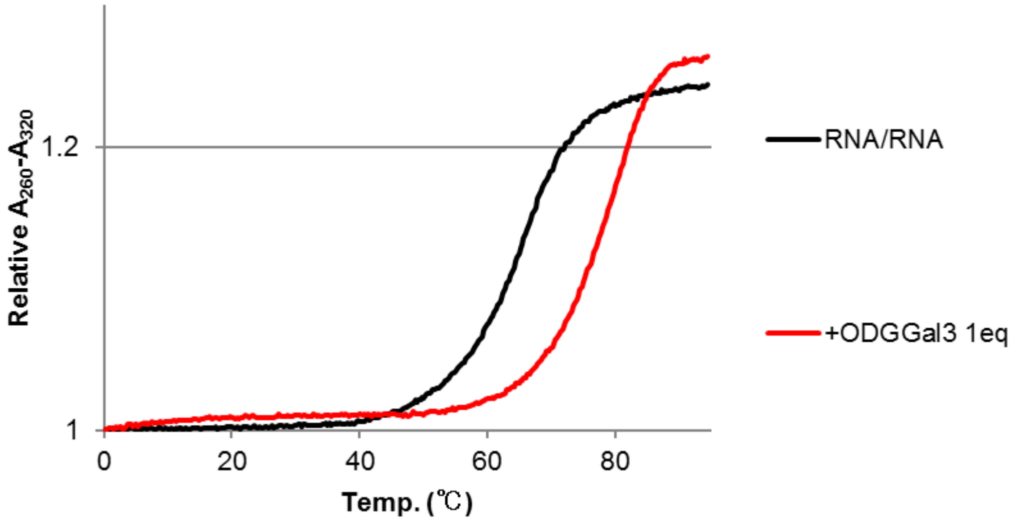


Figure S3. UV melting curves of RNA/RNA duplex CGCGAAUUCGCG/CGCGAAUUCGCG in the absence and presence of ODGGal3.

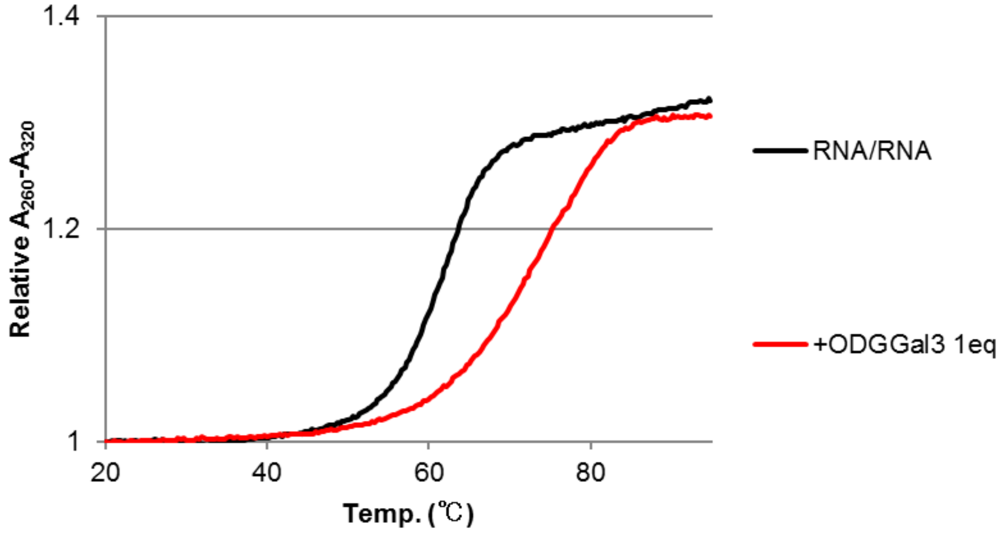


Figure S4. UV melting curves of RNA/RNA duplex $(CAGU)_3/(ACUG)_3$ in the absence and presence of ODGGal3.

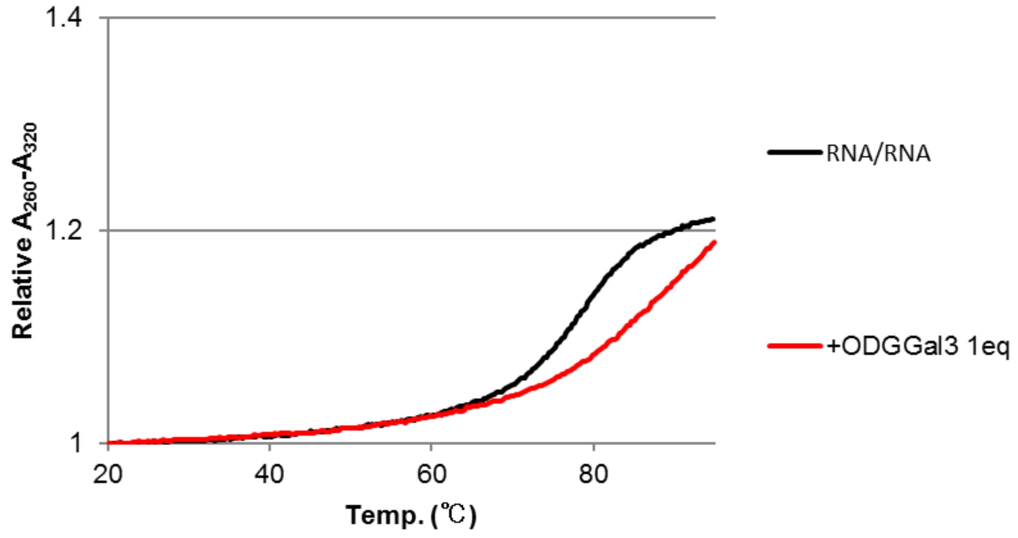


Figure S5. UV melting curves of RNA/RNA duplex AACCCGCGGGUU/AACCCGCGGGUU in the absence and presence of ODGGal3.

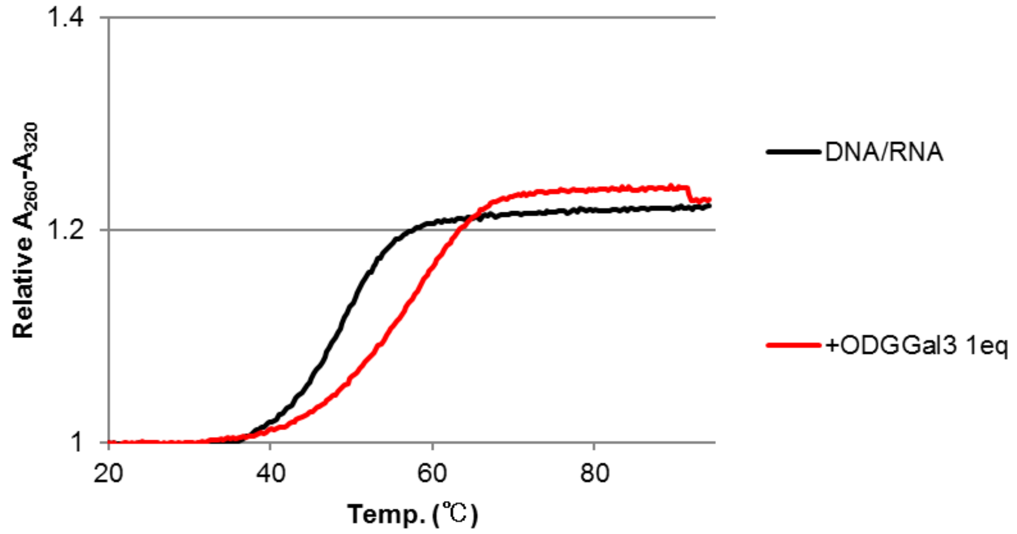


Figure S6. UV melting curves of DNA/RNA hybrid $(cagt)_3/(ACUG)_3$ in the absence and presence of ODGGal3.

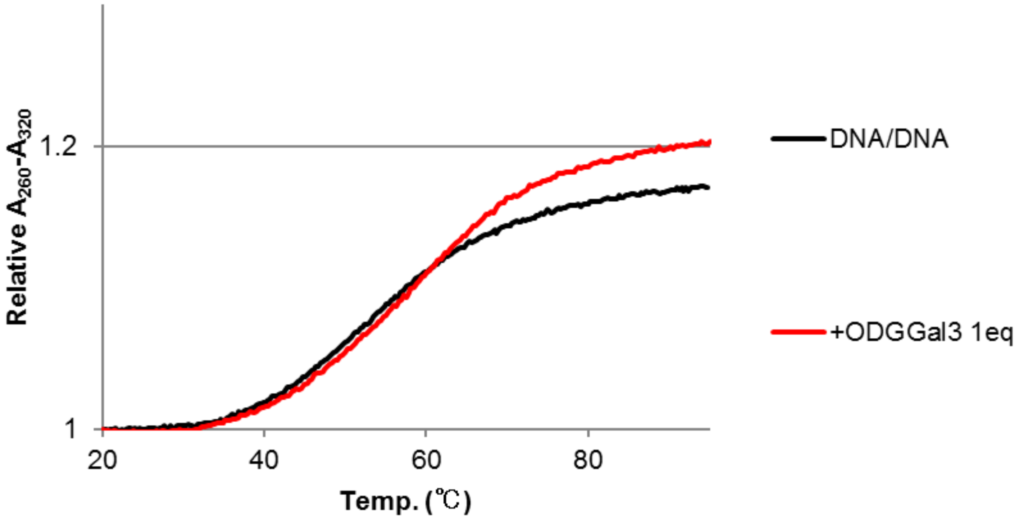


Figure S7. UV melting curves of DNA/DNA duplex cgcgaattcgcg/ cgcgaattcgcg in the absence and presence of ODGGal3.

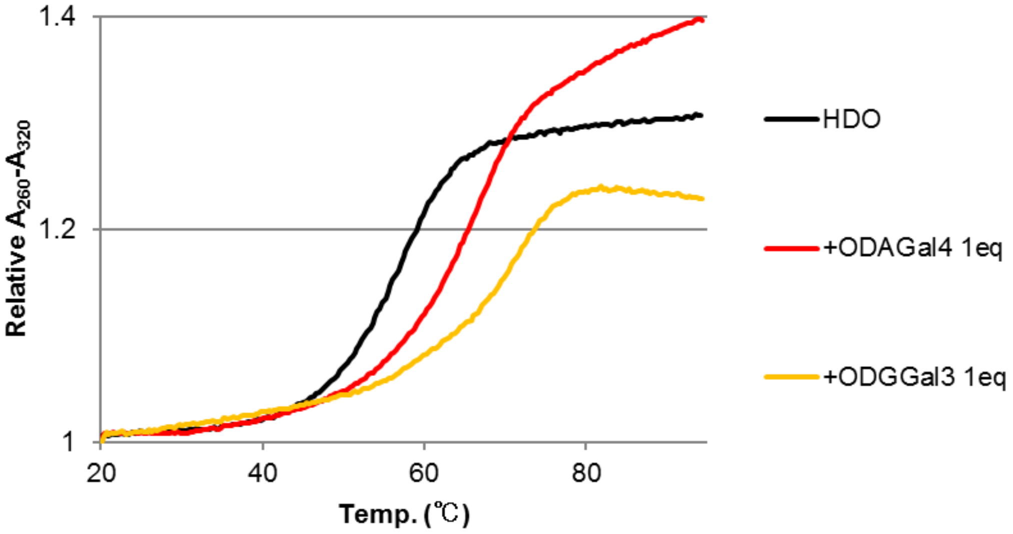


Figure S8. UV melting curves of the HDO in the absence and presence of ODAGal4 or ODGGal3.

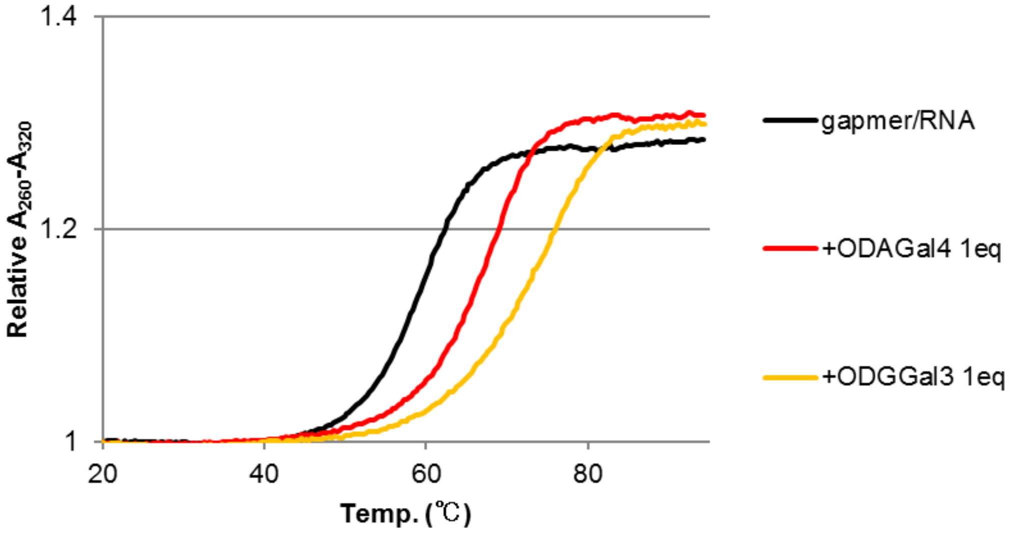


Figure S9. UV melting curves of the gapmer/RNA in the absence and presence of ODAGal4 or ODGGal3.

Table S1 $K_d$ values of ODGGal3 for their binding to FAM-labbeled 12mer nucleic acid duplexes at 20 °C in 10 mM phosphate buffer containing 100 mM NaCl and 0.2% Tween 20, pH 7.0. Base sequences are as follows: Entry 1: FAM-CGCGAAUUCGCG/FAM-CGCGAAUUCGCG, Entry 2: $(CAGU)_3$/FAM-$(ACUG)_3$, Entry3: FAM-AACCCGCGGGUU/FAM-AACCCGCGGGUU, Entry 4: $(cagt)_3$/FAM-$(ACUG)_3$, Entry 5: FAM-cgcgaattcgcg/FAM-cgcgaattcgcg. B (capital) = RNA; b (lower case) = DNA. These experiments were conducted according to the method described in our previous report.[11]

| Entry | duplex | $K_d/10^{-6}$ M |
|---|---|---|
| 1 | RNA/RNA | 0.16 ± 0.05 |
| 2 | RNA/RNA | 0.049 ± 0.008 |
| 3 | RNA/RNA | 0.15 ± 0.03 |
| 4 | DNA/RNA | 0.13 ± 0.03 |
| 5 | DNA/DNA | ND |

ND: not detectable

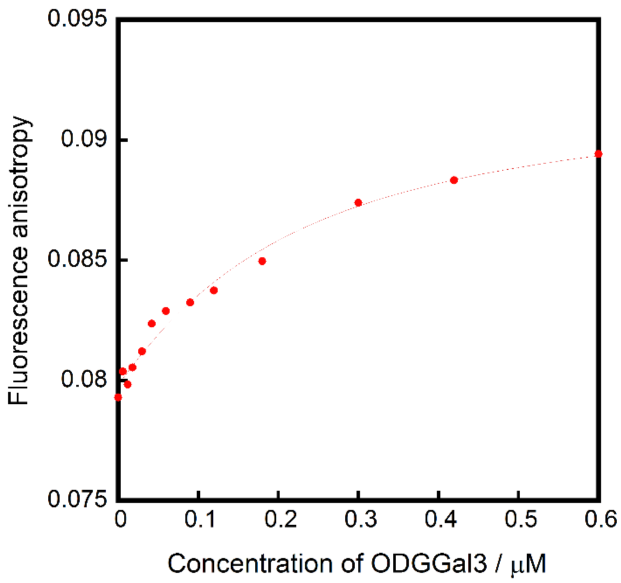


Fig. S10. Fluorescence anisotropy of 100 nM of $(FAM\text{-}CGCGAAUUCGCG)_2$ was titrated by increasing concentration of ODGGal3.

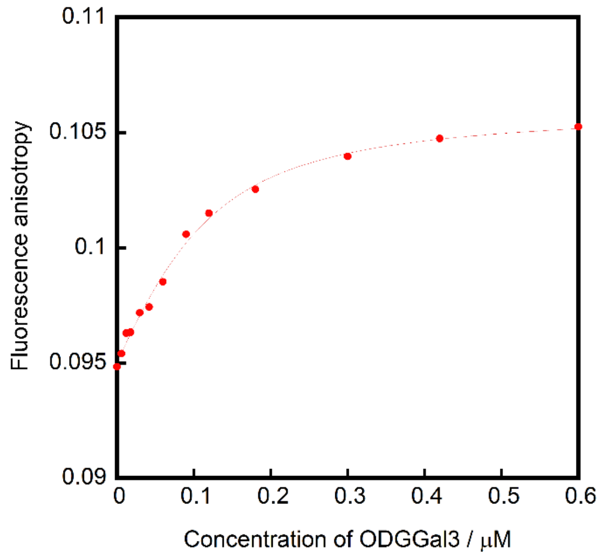


Fig. S11. Fluorescence anisotropy of 100 nM of $(CAGU)_3$/FAM-$(ACUG)_3$ was titrated by increasing concentration of ODGGal3.

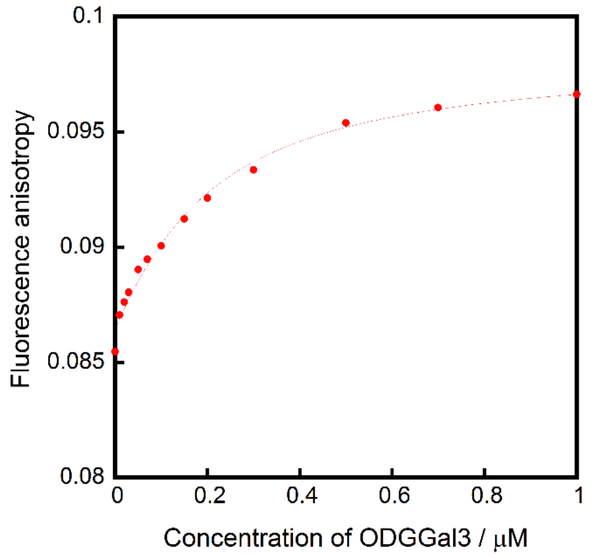


Fig. S12. Fluorescence anisotropy of 100 nM of FAM-AACCCGCGGGUU/FAM-AACCCGCGGGUU was titrated by increasing concentration of ODGGal3.

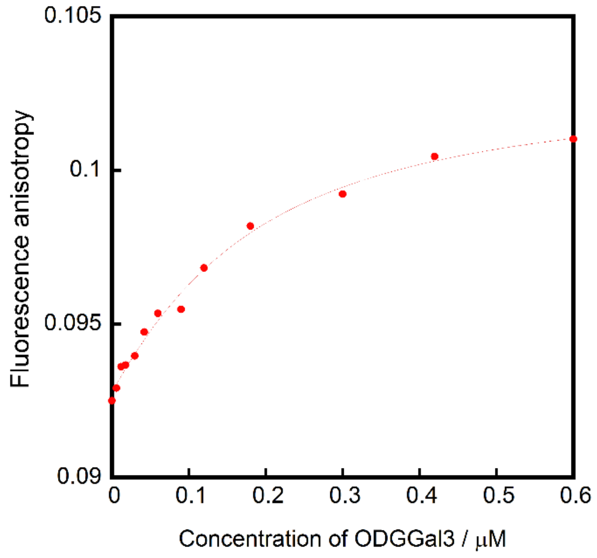


Fig. S13. Fluorescence anisotropy of 100 nM of $(cagt)_3$/FAM-$(ACUG)_3$ was titrated by increasing concentration of ODGGal3.

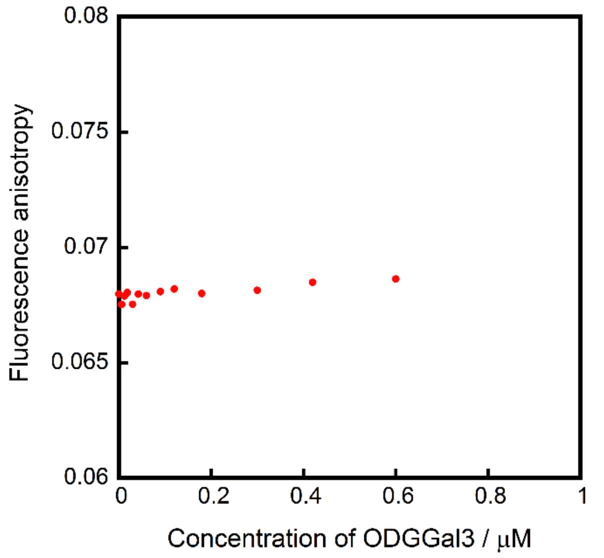


Fig. S14. Fluorescence anisotropy of 100 nM of FAM-cgcgaattcgcg/FAM-cgcgaattcgcg was titrated by increasing concentration of ODGGal3.

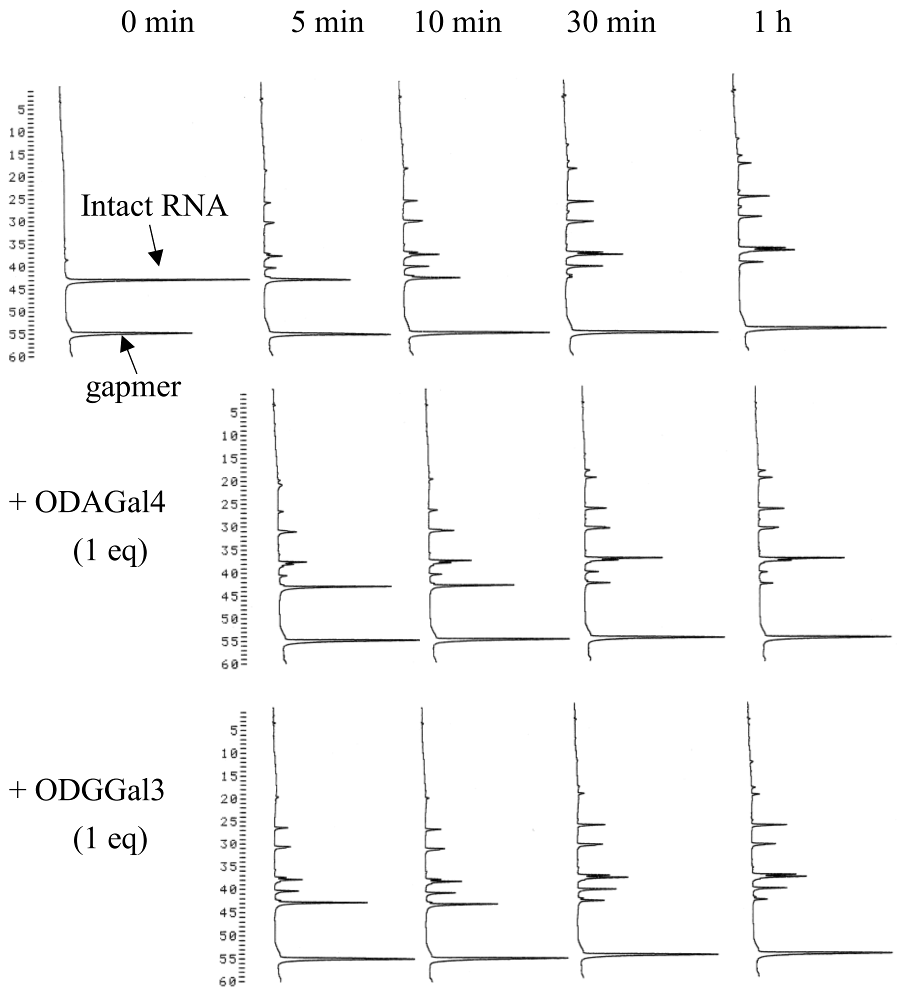


Figure S15. HPLC profiles of the gapmer (4 μM)/RNA (4 μM) after treatment with 40U/mL of RNase H over 5-60 min at 37 °C in the absence or presence of cationic oligosaccharides.

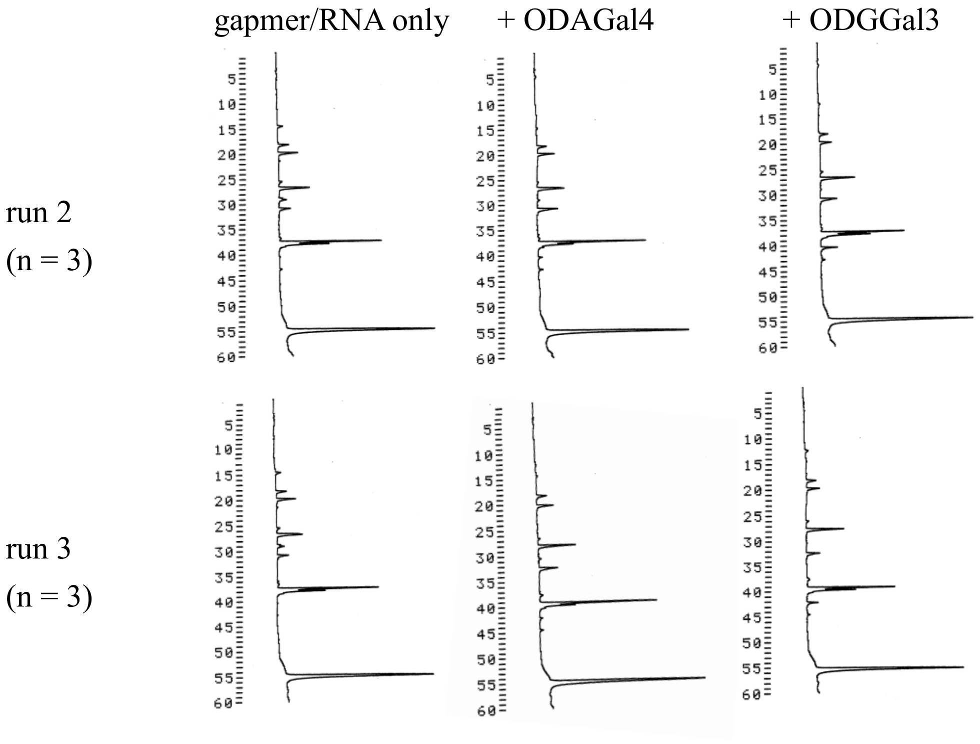


Figure S16. HPLC profiles of the gapmer (4 µM)/RNA (4 µM) after treatment with 40U/mL of RNase H over 1 h at 37 °C in the absence or presence of cationic oligosaccharides.

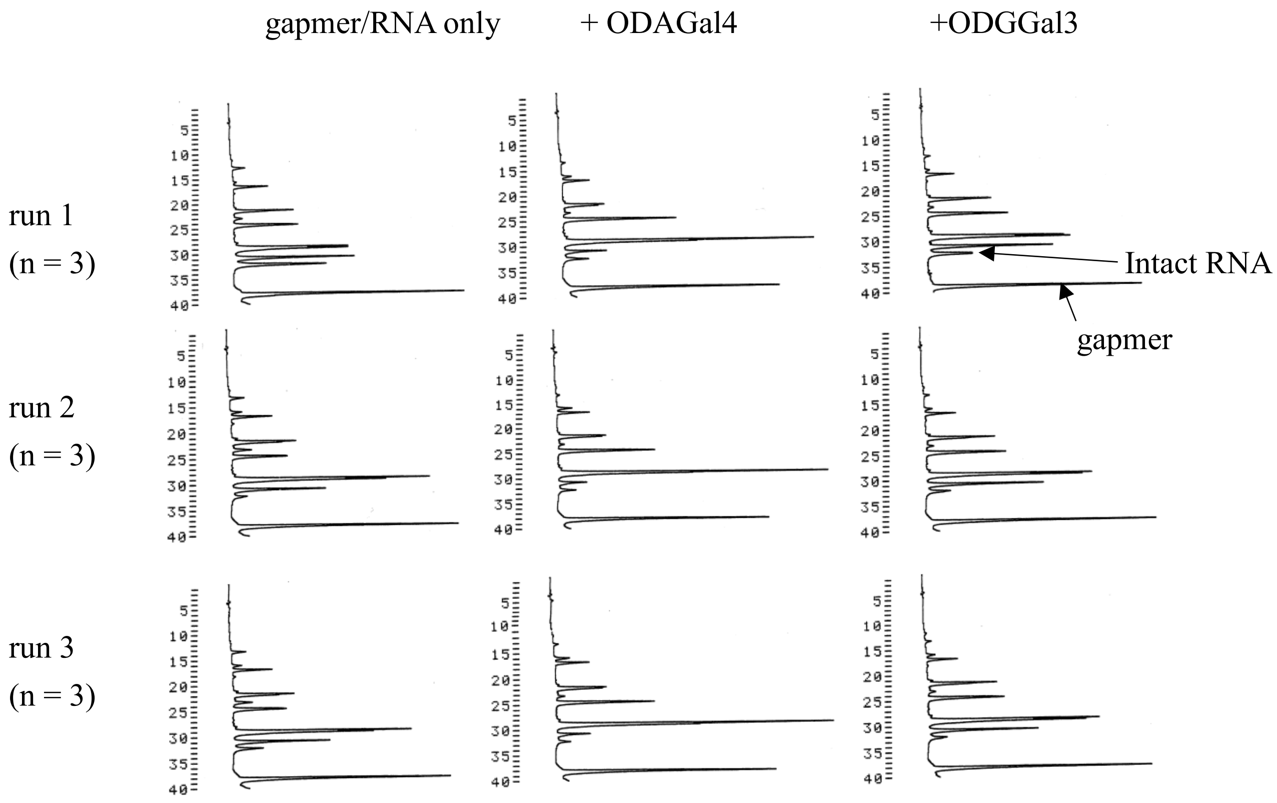


Figure S17. HPLC profiles of the gapmer (4 μM)/RNA (12 μM) after treatment with 40U/mL of RNase H over 1 h at 37 °C in the absence or presence of cationic oligosaccharides.

**Conditions for measurement of CD spectra.**

CD spectra were recorded with a Jasco J-820 using a 1 mm cell at wavelength of 320 to 200 nm. All the experiments were conducted in 10 mM phosphate buffer containing 100 mM NaCl at pH 7.0 and the concentration of the duplex and the cationic oligosaccharides was 5 μM. The following instrument settings were used: Resolution, 0.1 nm; Band width, 2.0 nm; response, 1 sec; speed, 100 nm/min; accumulation, 10.

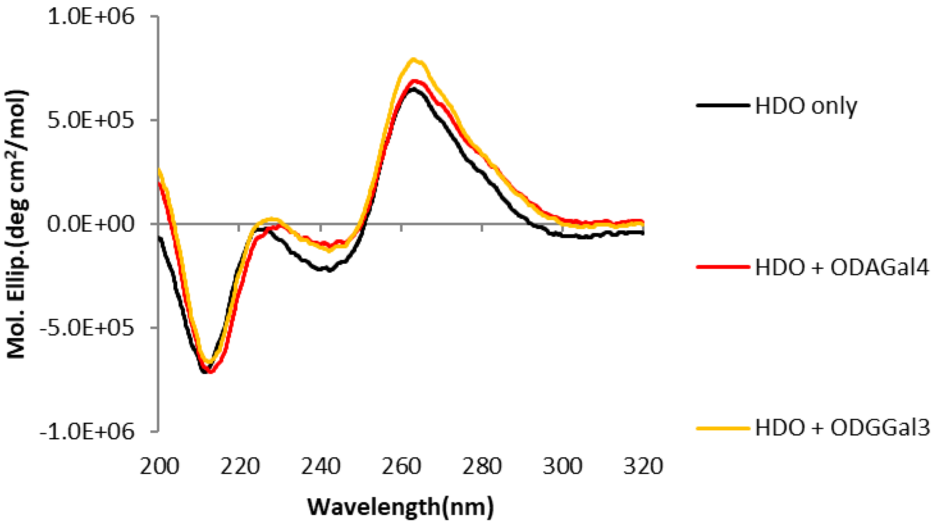


Figure S18. CD spectra of the HDO in the absence and presence of ODAGal4 or ODGGal3.
